# Aronia Upregulates Myogenic Differentiation and Augments Muscle Mass and Function Through Muscle Metabolism

**DOI:** 10.3389/fnut.2021.753643

**Published:** 2021-11-23

**Authors:** Chae-Eun Yun, Hyun-Kyung So, Tuan Anh Vuong, Myung Woo Na, Subin Anh, Hyo-Keun Lee, Ki Hyun Kim, Jong-Sun Kang, Gyu-Un Bae, Sang-Jin Lee

**Affiliations:** ^1^Department of Molecular Cell Biology, Single Cell Network Research Center, Sungkyunkwan University School of Medicine, Suwon, South Korea; ^2^Single Cell Network Research Center, Sungkyunkwan University School of Medicine, Suwon, South Korea; ^3^Research Institute of Aging Related Disease, AniMusCure Inc., Suwon, South Korea; ^4^School of Pharmacy, Sungkyunkwan University, Suwon, South Korea; ^5^Gyeonwoo Korean Medical Center, Seoul, South Korea; ^6^Drug Information Research Institute, College of Pharmacy, Sookmyung Women's University, Seoul, South Korea

**Keywords:** *aronia melanocarpa*, muscle atrophy, muscle differentiation, muscle mass and function, myofiber types

## Abstract

Black chokeberry or aronia (the fruit of *Aronia melanocarpa*) has been reported to having pharmacological activities against metabolic syndrome, such as hypertension, obesity, diabetes, and pro-inflammatory conditions. However, the effects of aronia on myogenic differentiation and muscle homoeostasis are uncharacterized. In this study, we investigated the effects of aronia (black chokeberry) on myogenic differentiation and muscle metabolic functions in young mice. Aronia extract (AR) promotes myogenic differentiation and elevates the formation of multinucleated myotubes through Akt activation. AR protects dexamethasone (DEX)-induced myotube atrophy through inhibition of muscle-specific ubiquitin ligases mediated by Akt activation. The treatment with AR increases muscle mass and strength in mice without cardiac hypertrophy. AR treatment enhances both oxidative and glycolytic myofibers and muscle metabolism with elevated mitochondrial genes and glucose metabolism-related genes. Furthermore, AR-fed muscle fibers display increased levels of total OxPHOS and myoglobin proteins. Taken together, AR enhances myogenic differentiation and improves muscle mass and function, suggesting that AR has a promising potential as a nutraceutical remedy to intervene in muscle weakness and atrophy.

## Introduction

In aging, skeletal muscles undergo a progressive decline in mass, strength, and functionality related to a condition called aging-related sarcopenia (1). Sarcopenia represents a risk factor for frailty, the loss of independence, and physical disability in the elderly (2). Since skeletal muscle constitutes about 40% of body mass in the healthy young person and contributes to body metabolic health, declines in muscle mass, and functionality will have consequences in the incidence of secondary aging-related diseases, such as metabolic syndrome, cardiovascular diseases, or chronic inflammation (3, 4). These secondary diseases will further exacerbate muscle loss, contributing to the increase in morbidity and mortality (5). Thus, the prevention of sarcopenia through enhancing muscle mass and function appears to be critical for the extension of a healthy life span in the elderly population. Multiple mechanisms, such as declines in mitochondrial function and muscle regenerative capacity or neuromuscular dysfunction, have been implicated in diverse muscle wasting conditions, such as sarcopenia (6–9). Thus, improving mitochondrial function, muscle regeneration, and motor neuron function is critical to prevent muscle wasting related to diverse conditions. Much attention has been paid to develop therapeutic tools to prevent muscle wasting and so far, the most effective intervention appears to be exercise (10). One of the major mechanisms of exercise is improving mitochondrial biogenesis and function through a transcription coactivator, called peroxisome proliferator-activated receptor gamma coactivator 1-alpha (PGC-1α). The increase in mitochondrial function through exercise or mimetics can protect muscle atrophy and weakness caused by aging or other conditions, such as denervation (11). Improved muscle stem cell function has been also associated with the protective effect of exercise-mediated mechanisms (12).

Other pathways more directly linked to the control of muscle mass are IGF1/Akt/mTOR and myostatin-Smad2/3 pathways controlling protein synthesis and degradation, respectively (13, 14). In diverse condition-related sarcopenia, the imbalance in this protein metabolism is one of the major causative mechanisms (15, 16). The excessive protein degradation relative to protein synthesis causes muscle atrophy related to a variety of conditions, such as starvation, denervation, cancer cachexia, and aging (11, 14, 17, 18). Two major protein breakdown pathways, the proteasomal and the autophagic-lysosomal pathways, are activated during muscle atrophy and variably contribute to the loss of muscle mass (19). Two muscle-specific E3 ligases, muscle RING finger containing protein 1 (MuRF1) and muscle atrophy F-box protein (Atrogin-1), are associated with the muscle protein degradation pathway (20–22). These E3 ligases are regulated by transcription factors, such as Forkhead box O3 (FoxO3), which in turn is negatively regulated by Akt signaling (19, 22, 23). On the other hand, Akt/mammalian target of rapamycin (mTOR) signaling promotes protein synthesis leading to muscle hypertrophy (24). Akt can repress the activity of FoxO transcription factors by phosphorylation and consequently the expression of MuRF1 and Atrogin-1 (25). Thus, Akt/mTOR signaling attenuates protein degradation by blocking FoxO action and increases protein synthesis through S6K activation, leading to muscle hypertrophy.

Black chokeberry or aronia (the fruit of *Aronia melanocarpa, A. melanocarpa*) is a shrub of the *Rosaceae* family, which is a plant native to North America, and was transferred to Europe about a century ago (4, 26). Aronia is traditionally used by Potawatomi Native Americans to cure colds and is also used as a tonic and adaptogen activity (27). Aronia has various biological activities based on phenolic compounds, such as anthocyanins, flavonols, flavanols, proanthocyanidins, and phenolic acids (28, 29). Aronia and its products have a great health-promoting potential related to reducing risk factors of metabolic syndrome, such as dyslipidemia, hypertension, obesity, glucose metabolism disorders, pro-inflammatory conditions, and thrombosis (4, 30–34). In addition, it is shown to have effects to inhibit the development of various types of cancers, such as leukemia, breast and intestinal cancer, and cancer stem cells (33, 35). In this study, we investigated the effects of aronia extract (AR) on myoblast differentiation and myotube atrophy triggered by dexamethasone (DEX). In addition, the effect of AR on muscle function and metabolism in mice was determined.

## Materials and Methods

### Reagents

Wild-type C57BL/6 male mice were purchased from (Orient-Bio, Seongnam, Korea). Fetal bovine serum (FBS), horse serum (HS), and Dulbecco modified Eagle's medium (DMEM) were purchased from Thermo Scientific (Waltham, MA, USA). 3-(4,5-dimethylthiazol-2-yl)-2,5-diphenyltetrazolium bromide (MTT), DEX, and all other chemicals were from Sigma-Aldrich (St. Louis, MO, USA). Antibodies were purchased as follows: myosin heavy chain (MHC, Developmental Studies Hybridoma Bank (DSHB), Iowa, IA, USA) Myogenin, Myoglobin, total-OxPHOS (Abcam, Cambridge, MA, USA), total-Akt, phospho-Akt, phospho-mTOR, mTOR, phospho-S6K, S6K (Cell Signaling Technology, Beverly, MA, USA), MuRF1, Atrogin-1, HSP90 (Santa Cruz Biotechnology, Santa Cruz, CA, USA), and β-tubulin (Zymed, South San Francisco, CA, USA).

### Aronia Material and Extraction Procedure

The fresh fruits of *A. melanocarpa* were collected at Danyang-gun, Chungcheongbuk-do, Korea, in August 2016 and were identified by one of the authors (K. H. Kim). A voucher specimen (SKKU AR-2016-08) has been deposited in the herbarium of the School of Pharmacy, Sungkyunkwan University, Suwon, Korea. Dried fruits (350 g) of *A. melanocarpa* were extracted with 80% MeOH for 3 days twice at room temperature. The extracts were then filtered, and the filtrate was concentrated under vacuum pressure, generating an AR (21.8 g). The AR was stored at −20°C until use.

### Animal Studies

All animal experiments were approved by the Institutional Animal Care and Research Advisory Committee at Sungkyunkwan University School of Medicine (SUSM) and complied with the regulations of the institutional ethics committee. All mice were maintained at 23°C with a 12:12 light-dark cycle and free access to food and water. To examine the effect of AR, these mice were orally administered a daily dose of 3.3 mg/kg AR for 8 weeks (8-month-old mice). Control mice were administrated the same amount of vehicle (dimethylsulfoxide, DMSO) dissolved in saline. All animals were sacrificed after fasting for 6 h with *ad libitum* to water.

### Cell Culture

C2C12 myoblasts were cultured as previously described (36). They were grown in Dulbecco's Modified Eagle Medium high glucose (DMEM; Thermo Scientific, Waltham, MA, USA) containing 15% FBS (growth medium, GM), 10 units/ml penicillin, and 10 μg/ml streptomycin (Welgene, Daegu, Korea) at 37°C, 5% CO_2_. To induce differentiation of C2C12 myoblasts, cells at near confluence were changed growth medium into DMEM containing 2% HS (differentiation medium, DM), and myotube formation was observed at 2 or 3 days after differentiation. For the DEX-induced atrophy study, C2C12 cells were induced to differentiate in DM for 3 days (D3), followed by the treatment with 100 μM DEX along with vehicle DMSO or AR for an additional 1 day (D4) (37).

### Western Blotting and Immunostaining

Western blot analysis was performed as previously described (38). Briefly, cells were lysed in cell extraction buffer (10 mM Tris-HCl, pH 8.0, 150 mM NaCl, 1 mM ethylenediaminetetraacetic acid (EDTA), and 1% Triton X-100) containing a complete protease inhibitor cocktail (Roche Diagnostics, Basel, Switzerland), followed by sodium dodecyl sulfate–polyacrylamide gel electrophoresis (SDS-PAGE) and incubation with primary and secondary antibodies.

Immunostaining for MHC expression was carried out as previously described (39). Briefly, the differentiated cultures were then immunostained for MHC antibodies and Alexa 568-conjugated secondary antibodies (Molecular Probes, Eugene, OR, USA). Images were captured and processed with a Nikon ECLIPSE TE-2000U microscope and NIS-Elements F software (Nikon, Tokyo, Japan). To analyze the efficiency of myotube formation, the MHC-positive myotubes containing two to five, or six or more nuclei, were quantified at least three times and measured using ImageJ software.

### Cryosections, Staining Analysis, and Fiber Size Measurement

Muscle tissue was embedded in Tissue-Tek OCT Compound (Sakura Finetek, Nagano, Japan), and 7 mm thick serial sections for staining were cut using a cryomicrotome. To analyze the nicotinamide adenine dinucleotide (NADH) dehydrogenase activity, we dried the sectioned tissues for 10 min at room temperature and incubated them in 0.9 mM NADH and 1.5 mM nitro blue tetrazolium (NBT; Sigma-Aldrich) in 3.5 mM phosphate buffer (pH 7.4) for 30 min. To analyze the succinate dehydrogenase (SDH) activity, we incubated the sections for 30 min in 50 mM sodium succinate and 0.3 mM nitro blue tetrazolium in 114 mM phosphate buffer containing K-EGTA (Sigma-Aldrich). To analyze the glycerol-3-phosphate dehydrogenase (GPDH) activity, we used the dried tissue samples and incubated them in 1.2 mM NBT, 2 mM phenazine methosulfate, and 1.86 mM glycerol phosphate in sodium phosphate buffer (pH 7.4) for 40 min.

Myh immunostaining of muscle tissue sections was performed in the sequence of fixation, permeation, and incubation with primary antibodies against MyhIIa and MyhIIb (DSHB) and laminin (Abcam). Images were captured with a Nikon ECLIPSE TE-2000U using NIS-Elements F software. Myofiber area was measured with ImageJ software. For muscle histology, the cryosections were stained with Mayer's hematoxylin and eosin (BBC Biomedical, McKinney, TX, USA). The images were captured using a Nikon ECLIPS TE-2000U.

### RNA Isolation and Quantitative Real-Time (RT)-PCR

Total RNA extraction and quantitative RT-PCR analysis were performed as previously described (40). Tissues were homogenized by FastPrepR-24 (MP Biomedicals, Santa Ana, CA, USA) and extracted using the easy-spin Total RNA Extract kit (iNtRON, Seongnam, Korea). Gene expression fold change was normalized against the expression of 18S ribosomal RNA. The sequences of the primers used in this study are provided in Table 1.

**Table 1:** The primers used in this study.

**Primer**		**Sequence**
MyhIIa	Forward	5'-GGCTTCAGGATTTGGTGGATAA-3'
	Reverse	5'-GGATCTTGCGGAACTTGGATAG-3'
MyhIIb	Forward	5'-GATTGACGTGGAGAGGTCTAAC-3'
	Reverse	5'-CCTGAGTTTCCTCGTACTTCTG-3'
MTCO	Forward	5'-CTACTATTCGGAGCCTGAGC-3'
	Reverse	5'-GCATGGGCAGTTACGATAAC-3'
SDHB	Forward	5'-ACCCCTTCTCTGTCTACCG-3'
	Reverse	5'-AATGCTCGCTTCTCCTTGTAG-3'
Myoglobin	Forward	5'-CACCATGGGGCTCAGTGATG-3'
	Reverse	5'-CTCAGCCCTGGAAGCCTAGC-3'
HK	Forward	5'-GCTGGAGGTTAAGAGAAGGATG-3'
	Reverse	5'-TGGAGTGGCACACACATAAG-3'
PK	Forward	5'-CATGCAGCACCTGATAGC-3'
	Reverse	5'-AGCTGCTGCTAAACACTTAT-3'
PFK	Forward	5'-ACCAGAGCACGTTTGTGTTAG-3'
	Reverse	5'-GGCGGACACTCAGGAATAAA-3'
MuRF1	Forward	5'-GAGAACCTGGAGAAGCAGCT-3'
	Reverse	5'-CCGCGGTTGGTCCAGTAG-3'
Atrogin-1	Forward	5'-CAACATTAACATGTGGGTGTAT-3'
	Reverse	5'-GTCACTCAGCCTCTGCATG-3'
18S rRNA	Forward	5'-AGGGGAGAGCGGGTAAGAGA-3'
	Reverse	5'-GGACAGGACTAGGCGGAACA-3'

### Grip Strength Test

Grip strength was measured using a grip strength meter (Bioseb, Pinellar Park, FL, USA). The animal was allowed to grab the grid with a fore and back limb through a blind test. The limbs are pulled gently with consistent force until the forelimb was detached from the grid. The maximal strength was recorded when the grid was detached. Each animal was tested in three trials.

### Statistical Analysis

Values are expressed as mean ± SD for *in vitro* systems or ± SEM for *in vivo* systems, as indicated in the figure legends. The statistical significance was calculated using either Student's *t*-test (unpaired, two-tailed) or ANOVA by *post-hoc* Tukey's tests for multiple comparisons. Differences were considered statistically significant at or under values of *P* < 0.05.

## Results

### AR Enhances Myoblast Differentiation Through Akt Activation

**Figure 1:**
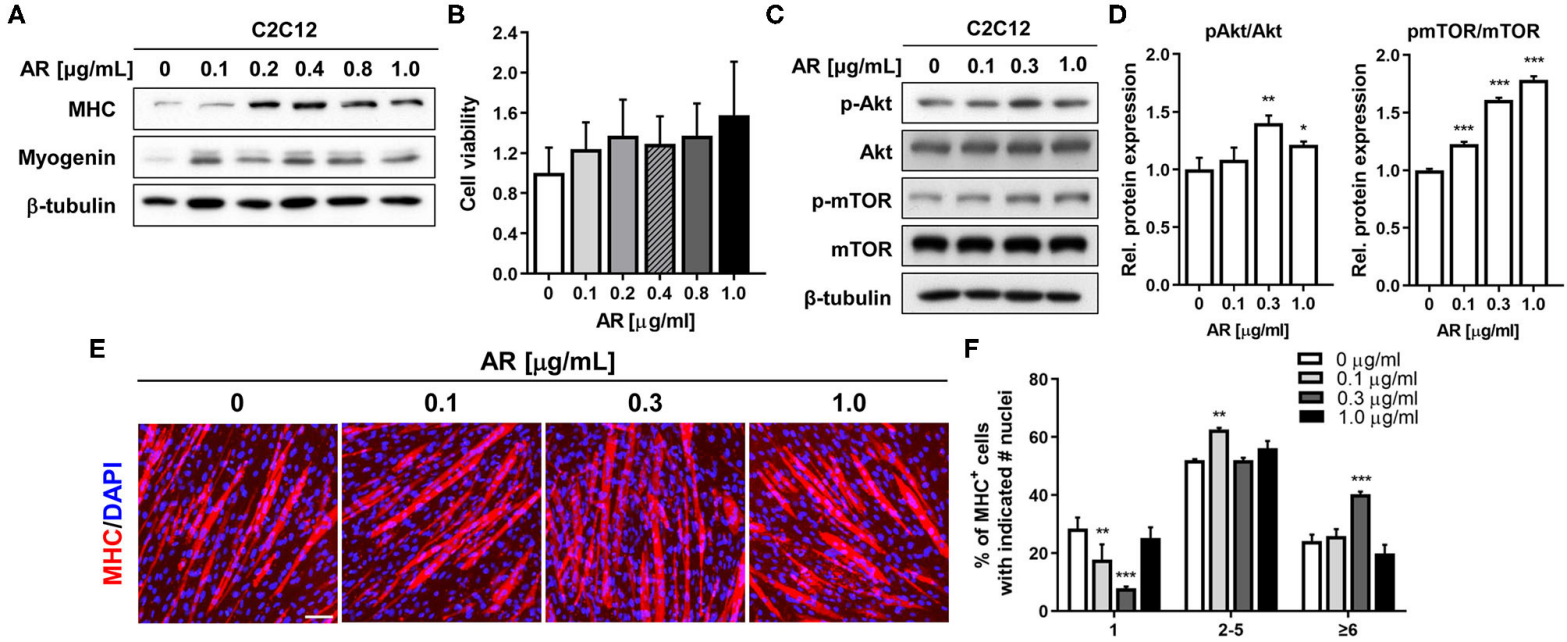
AR promotes myoblasts differentiation in C2C12 cells without cytotoxicity. **(A)** C2C12 myoblasts were induced to differentiate for 3 days in the presence of DMSO or the indicated concentration of AR in DM. The lysates were subjected to immunoblotting analysis with muscle-specific proteins, MHC, and Myogenin, β-Tubulin was used as a loading control. The experiment was repeated three times with similar results. **(B)** Cell viability was determined with an MTT assay. **(C)** Immunoblot analysis of C2C12 cells for the expression of the phosphorylated form of Akt and total Akt and β-tubulin as a loading control treated with vehicle or AR for D3. **(D)** Quantification of blots from three experiments similarly performed as shown in panel C. The signal intensity of pAkt and pmTOR was quantified, and the values were normalized to total Akt and mTOR, respectively. **(E)** Immunostaining for MHC expression (red) and DAPI (blue) staining to visualize nuclei. C2C12 myoblasts were induced to differentiation for 3 days in the presence of DMSO or the indicated concentration of AR in DM. Scale bar: 50 μm. **(F)** The MHC-positive myocytes shown in panel E were quantified as a number of nuclei per myotube. The one-way ANOVA analysis with Tukey test. Data represent means ± SD. ^*^P < 0.05 and ^**^P < 0.01. AR, Aronia extract; DMSO, DM, differentiation medium; MHC, myosin heavy chain; DAPI.

To examine the effect of AR on myogenic differentiation, C2C12 myoblasts were induced to differentiate for 3 days (D3) in the presence of vehicle DMSO or AR with the indicated concentration ranging from 0.1 to 1 μg/ml in DM and subjected to the assessment of myoblast differentiation by immunoblotting analysis. The treatment with AR increased the expression levels of MHC and Myogenin in a dose-sensitive fashion and peaked at the concentration between 0.2 and 0.4 μg/ml (Figure 1A). This slight decrease at higher AR concentration was not due to any cytotoxicity as assessed by MTT assay (Figure 1B). To explore the molecular regulatory pathways of AR-mediated myogenic promotion, C2C12 myoblasts were treated with AR at the concentration of 0.1, 0.3, and 1.0 μg/ml for 48 h. Cell lysates were subjected to immunoblotting analysis for the active phosphorylated Akt (pAkt) to assess for the activation status of a promyogenic kinase Akt. The treatment of AR, especially 0.3 μg/ml concentration, increased the level of pAkt without altering the total Akt protein level (Figures 1C,D). In addition, AR treatment induced the phosphorylation of mTOR, a downstream target of Akt, in a dose-dependent manner (Figures 1C,D). These results indicate that AR promotes myogenic differentiation with enhanced Akt activation.

To confirm the promyogenic effects of AR, myotube formation was assessed by MHC immunostaining. As a result, AR treatment in C2C12 myoblasts elicited the formation of larger MHC-positive multinucleated myotubes, compared to control cells (Figure 1E). To quantify the myotube formation, MHC-positive myocytes were counted as mononucleate, myotubes containing two to five nuclei or containing six or more nuclei and plotted as a percentile (Figure 1F). The treatment with AR decreased the proportion of mononucleate myocytes, while it substantially elevated the proportion of larger myotubes containing six or more nuclei in a dose-sensitive manner. Our current data further support the positive effect of AR on myoblast differentiation at a morphological and at a biochemical level. AR can exert its promyogenic effect through Akt activation without overt cytotoxicity.

### AR Protects DEX-Induced Myotube Atrophy Through Activation of Akt Signaling

**Figure 2:**
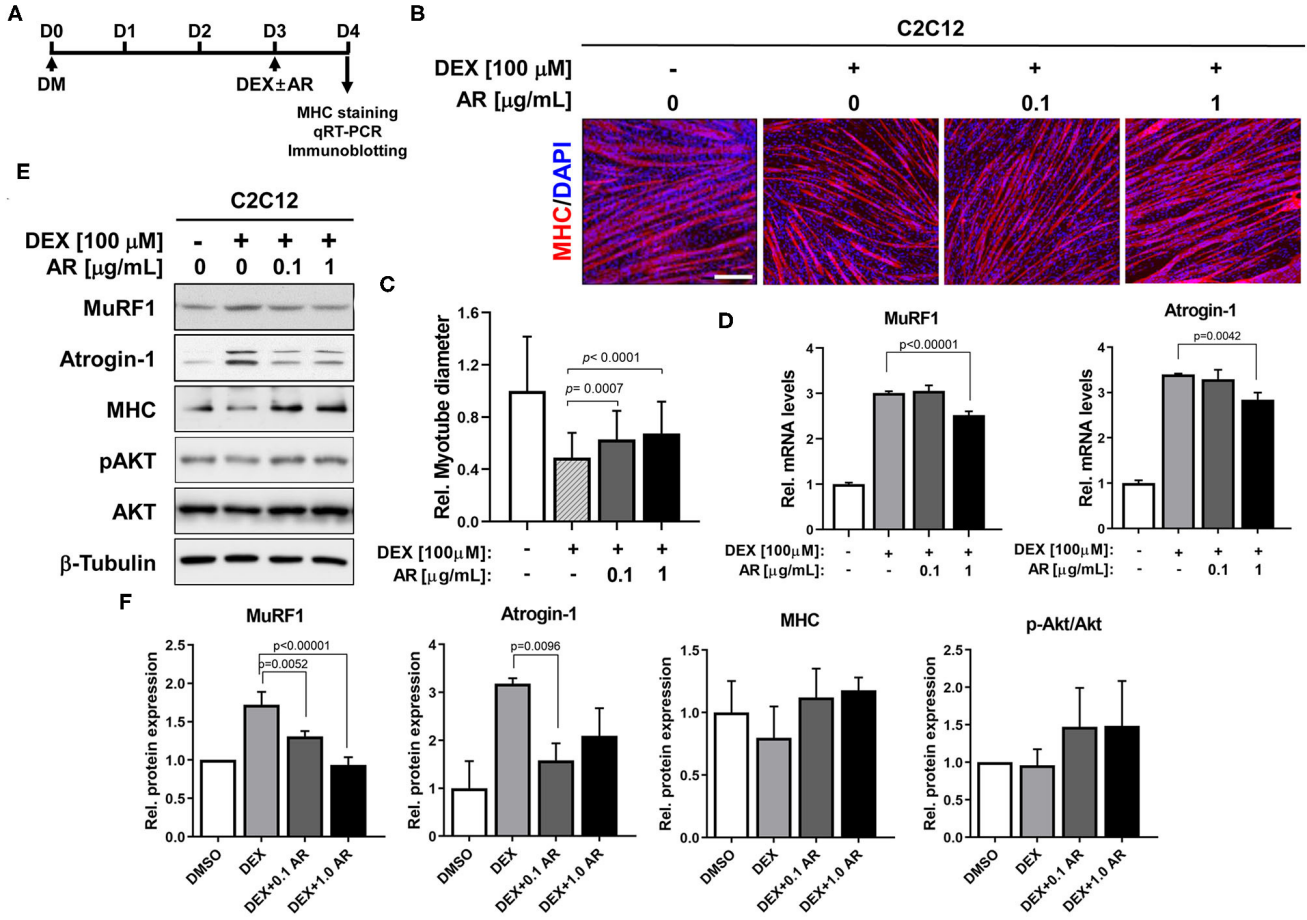
AR prevents DEX-induced muscle atrophy. **(A)** The procedure diagram for atrophy study. **(B)** Immunostaining for MHC expression (red) and DAPI (blue) staining to visualize nuclei. C2C12 myoblasts were induced to differentiate for 3 days in DM and treated with vehicle DMSO or indicated concentration of AR with DEX. Scale bar, 60 μm. **(C)** The relative diameter for panel B was quantified to show effect of AR from DEX-induced muscle atrophy. *N* = 123 myotubes/each sample. **(D)** qRT-PCR analysis for expression of MuRF1 and Atrogin-1 in C2C12 cells of the vehicle or DEX-treated AR. **(E)** Immunoblot analysis of C2C12 cells for the expression of MHC, MuRF1, Atrogin-1, pAkt and Akt, and β-tubulin as a loading control treated with vehicle or DEX-treated AR for D3. **(F)** Quantification of blots from three experiments similarly performed as shown in panel E. The signal intensity of MuRF1, Atrogin-1, and MHC was quantified, and the values were normalized to β-tubulin. The signal intensity of pAkt was quantified, normalized to total Akt. The one-way ANOVA analysis with Tukey test. AR, Aronia extract; DEX, dexamethasone; MHC, myosin heavy chain; DAPI.

The synthetic glucocorticoid DEX induces muscle-specific ubiquitin ligase expression contributing to muscle atrophic phenotypes with reduced myotube diameter (37). Akt activation is one of the key events to suppress the induction of muscle-specific ubiquitin ligase and muscle atrophy triggered by DEX (25). Since AR can activate Akt, the effects of AR at the concentration of 0.1 or 1.0 μg/ml on DEX-induced C2C12 myotube atrophy were examined. C2C12 cells were induced to differentiate in DM for 3 days (D3), followed by the treatment with DEX along with vehicle DMSO or AR for an additional 1 day (D4) (Figure 2A). Myotubes were then subjected to MHC immunostaining to access the thickness of myotubes. The treatment with DEX-elicited myotube atrophy, which was suppressed by AR treatment, was evident by the presence of larger multinucleated myotubes in AR-treated cultures (Figure 2B). The quantification of myotube diameter revealed that DEX treatment caused declines in myotube diameter, which was significantly recovered by AR treatment (Figure 2C). In addition, the qRT-PCR analysis showed that DEX treatment greatly elevated muscle-specific E3 ubiquitin ligases, MuRF1, and Atrogin-1 and AR treatment attenuated this induction (Figure 2D). To further define, the protein levels of muscle-specific proteins and ubiquitin ligases were determined by immunoblotting analysis. Consistent with qRT-PCR results, the treatment with AR in DEX-treated myotubes reduced the level of MuRF1 and Atrogin-1 proteins, compared to control (Figures 2E,F). The treatment of DEX elicited a reduction in MHC protein levels and the co-treatment of DEX with AR attenuated the decline in MHC proteins in myotubes. In addition, DEX-treated myotubes exhibited decreased levels of pAkt while AR treatment in DEX-treated myotubes abrogated this decrease. Taken together, these results suggest that AR protects DEX-induced myotube atrophy through inhibition of muscle-specific ubiquitin ligases mediated by Akt activation.

### AR Enhances Muscle Mass and Functions in Young Mice

**Figure 3:**
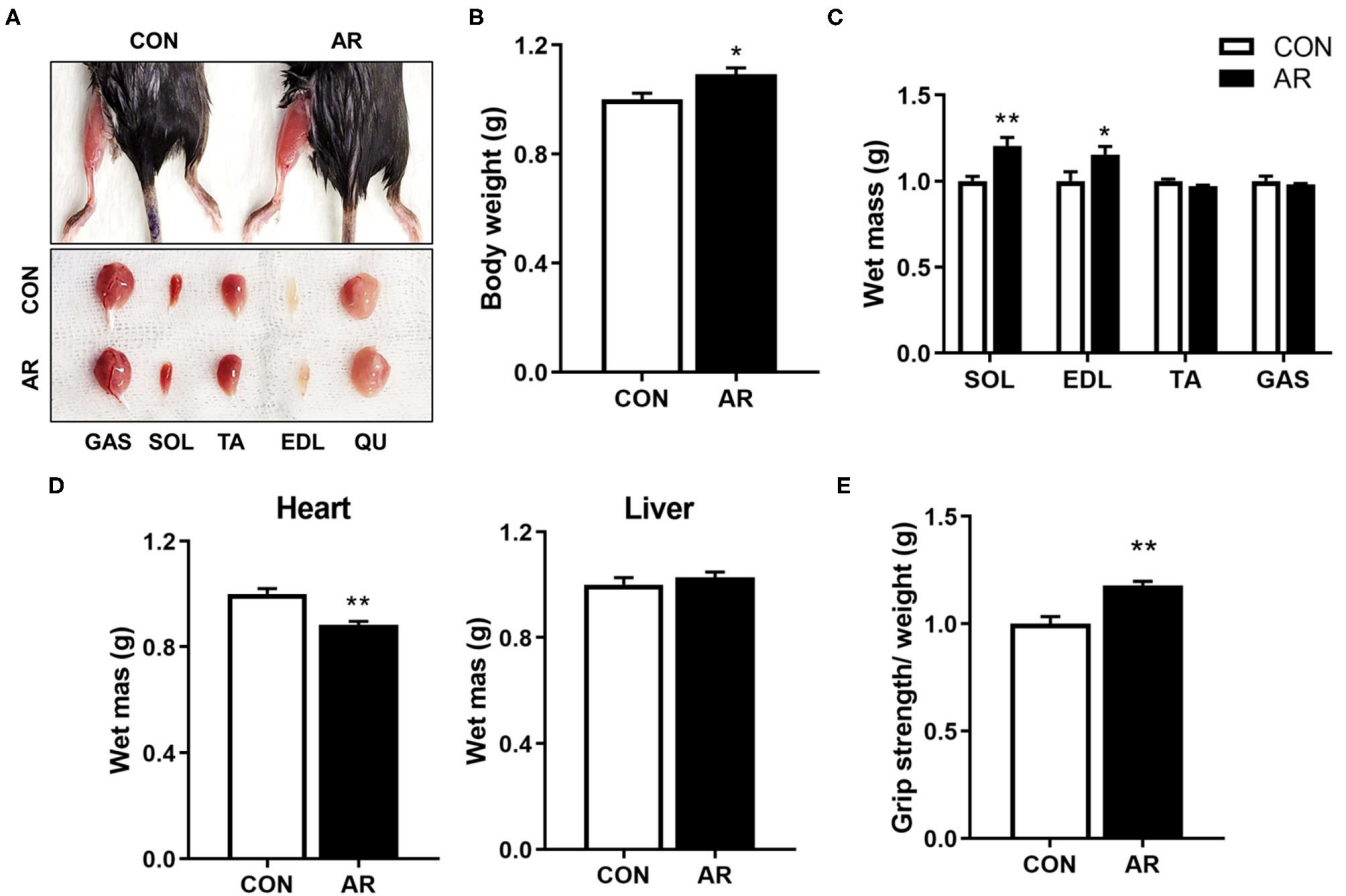
AR enhances muscle mass and strength. **(A)** Photographs of isolated muscle types from hindlimbs from 8-month-old mice ingested with control or 3.3 mg/kg AR for 8 weeks. **(B)** Body weight of 8-month-old control-fed and AR-fed mice for 8 weeks. **(C, D)** Weights of **(C)** four muscle types and **(D)** heart and liver from control or 3.3 mg/kg AR-ingested mice for 8 weeks, *n* = 3. **(E)** Grip strength depicted as the force (gram) that animals in each group pulled, n = 5. An unpaired two-tailed student's *t*-test. The data represent the mean ± SEM, ^*^P < 0.05 and ^**^P < 0.01. AR, Aronia extract.

To assess the *in vivo* effects of AR on muscles, 8-month-old-mice were fed with control or AR for 8 weeks, followed by muscle functional analysis and biopsy. The hindlimb muscles of AR-ingested mice appeared darker than those of control-fed mice, which can be readily detected in gastrocnemius (GAS) muscles among five hindlimb muscle groups, such as soleus (SOL), tibialis anterior (TA), extensor digitorum longus (EDL), and quadriceps (QU) (Figure 3A). AR treatment mildly but significantly elevated body weights, compared with the vehicle-treated group (Figure 3B). Among four hindlimb muscles, the weights of SOL and EDL muscles were increased an approximately 20.0 and 15.3% in AR-fed mice, compared to vehicle-fed mice, respectively (Figure 3C). The heart mass was slightly reduced but the liver mass was not changed (Figure 3D). One week prior to harvesting muscles, muscle strength was examined by measuring the grip strength. Mice fed with AR displayed approximately 14.5% increase in grip strength relative to control mice (Figure 3E). These data collectively suggest that AR treatment improves muscle mass and function without overt hypertrophic effect on the cardiac muscle.

### AR Increases Both Glycolytic and Oxidative Myofibers in Young Mice

**Figure 4:**
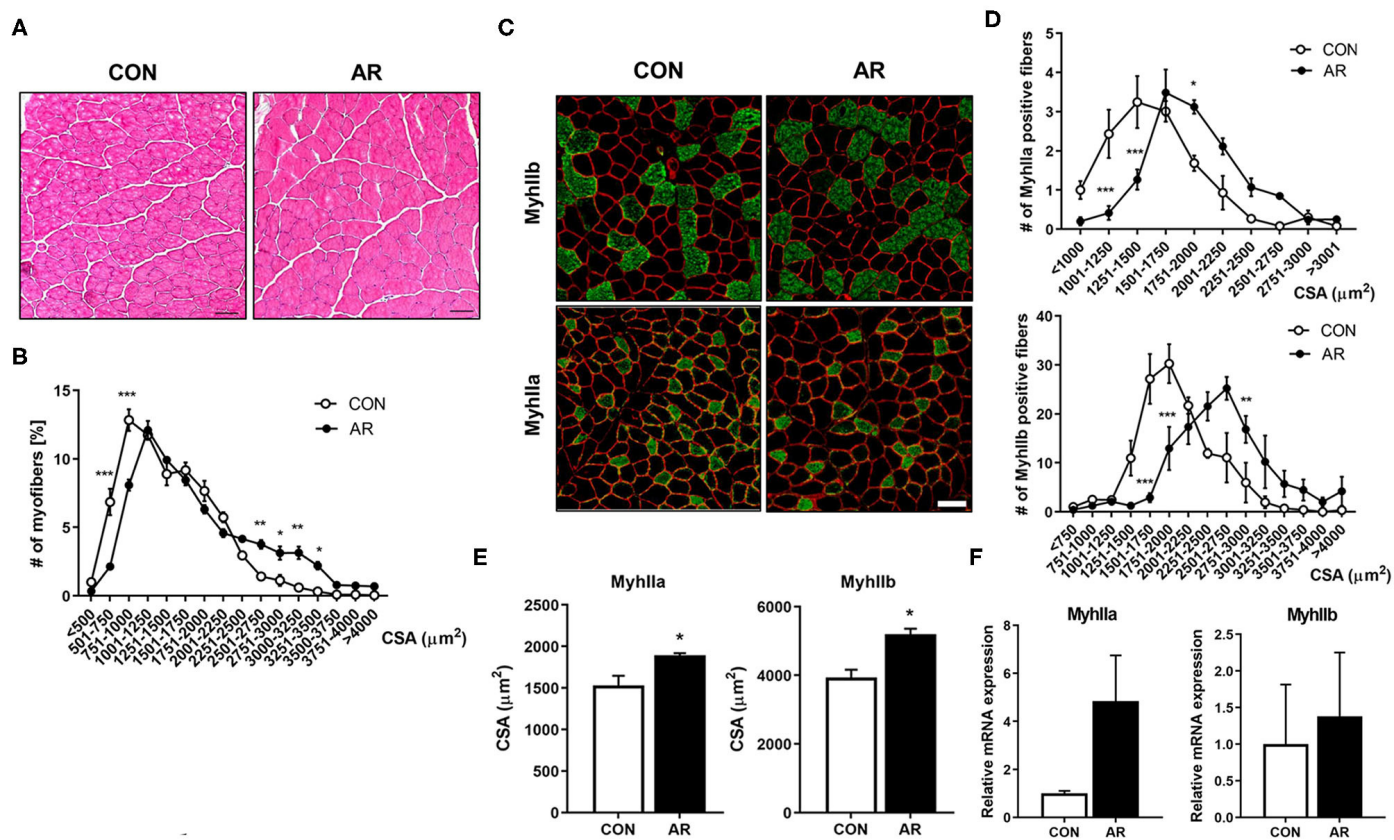
AR induces the formation of larger MyhIIa- and MyhIIb-positive myofibers. **(A)** Histological staining of TA muscles of 8-month-old mice ingested with control or 3.3 mg/kg AR for 8 weeks. H&E staining shows the structural recovery. Scale bar, 50 μm. **(B)** Quantification of the cross-sectional area of total myofibers in TA. **(C)** Immunostaining of MyhIIb (green), MyhIIa (green), and laminin (red) in the TA muscles of control or 3.3 mg/kg AR-ingested mice for 8 weeks. Scale bar, 50 μm. **(D, E)** Quantification of MyhIIb- and MyhIIa-positive myofibers in panel **(C)** (*n* = 3). **(F)** qRT-PCR analysis for expression of MyhIIa and MyhIIb in the TA muscles of control or 3.3 mg/kg AR-ingested mice for 8 weeks (*n* = 3). An unpaired two-tailed student's *t*-test. The data represent the mean ± SEM, ^*^P < 0.05, ^**^P < 0.01 and ^***^P < 0.001. AR, Aronia extract; TA, tibialis anterior.

To examine the detailed histology of muscles, control and AR-treated TA muscles were cryosectioned and subjected to hematoxylin and eosin staining, followed by the measurement of the cross-sectional area of myofibers (Figures 4A,B). AR-treated muscles had more lager myofibers, compared to the control-treatment muscles. To examine the effect of AR on myofiber types, muscle sections were analyzed by immunostaining with antibodies to MyhIIb and MyhIIa (Figure 4C). AR treatment elicited a shift toward larger MyhIIa- and MyhIIb-positive myofibers, compared to the vehicle treatment (Figures 4D,E). Consistently, qRT-PCR analysis of AR-treated muscles had elevated expression of MyhIIa and MyhIIb, compared to the control muscles (Figure 4F). These data suggest that AR treatment enhances MyhIIa and MyhIIb muscles.

### AR Improves Both Muscle Oxidative and Glycolytic Metabolism in Young Mice

**Figure 5:**
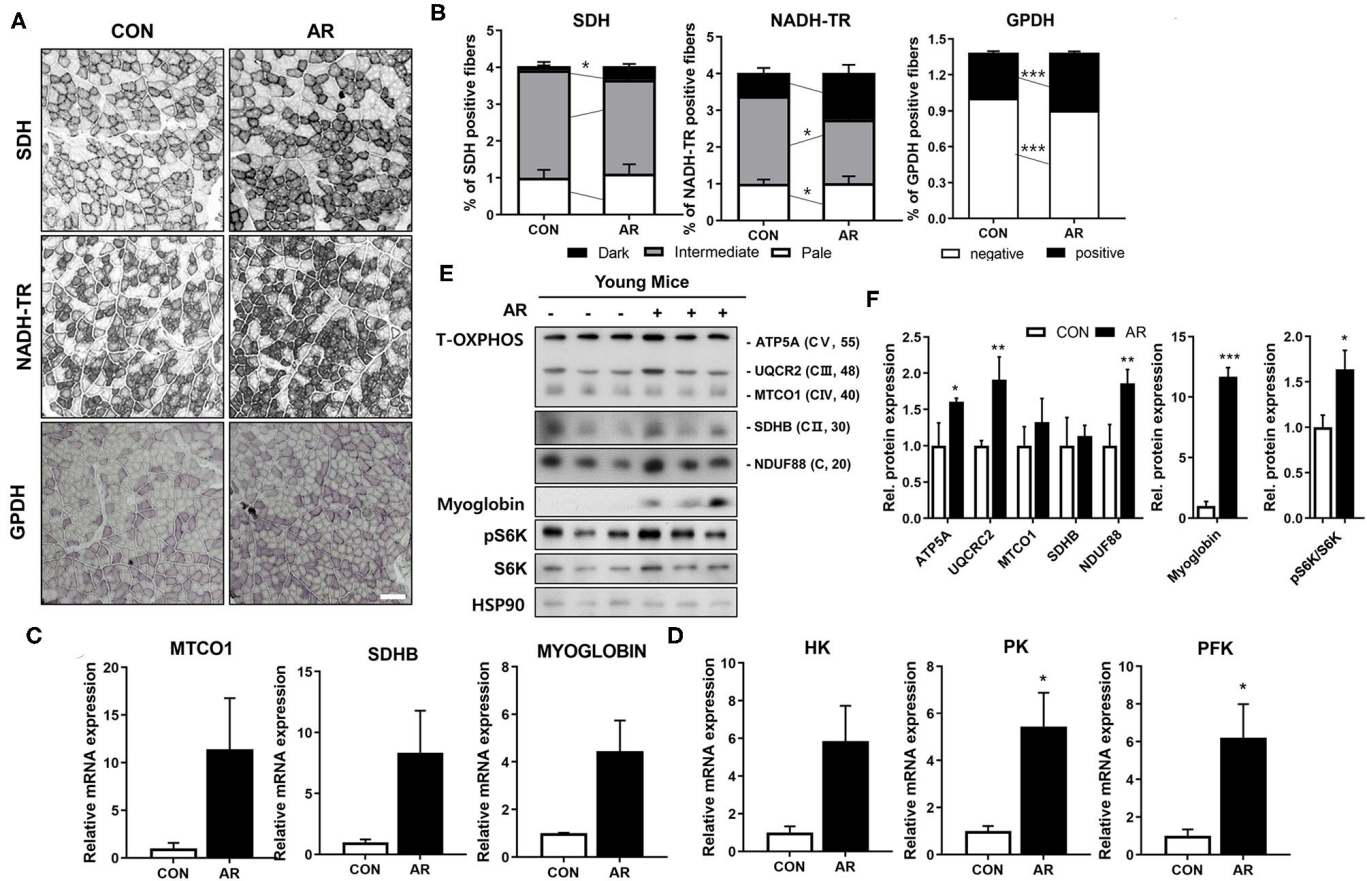
AR increases muscle metabolism in young mice. **(A)** Histochemical staining for SDH, NADH-TR and GPDH enzymatic activities in TA muscles. Scale bar, 50 μm. **(B)** The staining intensities of SDH and NADH-TR are quantified as three different grades (dark, intermediate, and pale) and plotted as a percentile, n = 3. An unpaired two-tailed student's *t*-test. **(C)** qRT-PCR analysis for expression of mitochondrial genes (MTCO1, SDHB) and myoglobin in TA muscles. **(D)** qRT-PCR analysis for expression of glucose metabolism-related genes in TA muscles. An unpaired two-tailed student's *t*-test. **(E)** Immunoblotting analysis for the expression of Total-OXPHOS, myoglobin and S6K in Qu muscles. **(F)** Quantification of the levels of total-OxPHOS proteins from panel E, *n* = 3. An unpaired two-tailed student's *t*-test. The data represent the mean ± SEM (*n* = 3). ^*^P < 0.05, ^**^P < 0.01 and ^***^P < 0.001. AR, Aronia extract; TA, tibialis anterior; SDH, succinate dehydrogenase; GPDH, glycerol-3-phosphate dehydrogenase; NADH-TR, nicotinamide adenine dinucleotide tetrazolium reductase.

Myofibers have different metabolic characteristics. Myofiber type IIa has higher mitochondrial content, compared to glycolytic myofiber type IIb (41, 42). Since AR substantially elevated both MyhIIa and MyhIIb expressions, we next examined the muscle metabolism by measuring activities of SDH and nicotinamide adenine dinucleotide tetrazolium reductase (NADH-TR) for muscle oxidative metabolism and GPDH for glycolytic metabolism. In agreement with the myofiber type staining, AR treatment elevated the proportion of myofibers with strong (dark) activities for SDH, NADH-TR, and GPDH, compared to the control muscles (Figures 5A,B), indicating that AR enhances both oxidative and glycolytic muscle metabolism. Consistently, AR-treated muscles expressed higher levels of mitochondrial genes (MTCO1, Sdhb), myoglobin, and glucose metabolism-related genes [hexokinase (HK), pyruvate kinase (PK), phosphofructokinase (PFK)], compared to the control muscles (Figures 5C,D). To further confirm, we examined the protein levels of total OXPHOS [ATP5A (CV), MTCO1 (CIII), SDHB (CII) and NDUF88 (CI)] proteins, myoglobin and S6K in Qu muscle tissues. In consistent with the RNA expression data, AR-treated muscles had elevated levels of total OXPHOS, myoglobin, and phosphorylated S6K proteins (Figures 5E,F). These data suggest that AR enhances the expression of muscle-specific genes and muscle metabolic genes, such as mitochondrial components.

## Discussion

Much attention has been paid to the discovery of effective pharmacological supplements to improve muscle mass and function in pathological conditions or the aging process. Recent advances in muscle biology led to new interests in pharmacological or nutraceutical treatment to prevent muscle atrophy and weakness. In this study, we investigate the effects of AR on myogenic differentiation and muscle function in mice. Our results demonstrate that AR elicits myoblast differentiation and the formation of multinucleated myotubes through Akt activation without overt cytotoxicity. The treatment of AR in DEX-induced atrophic myotubes restores myotube formation through suppression of muscle-specific ubiquitin ligases, likely mediated by Akt activation. In addition, AR treatment improves muscle mass and function through elevating the expression of muscle-specific genes and muscle metabolic genes without overt hypertrophic effect on the cardiac muscle.

**Figure 6:**
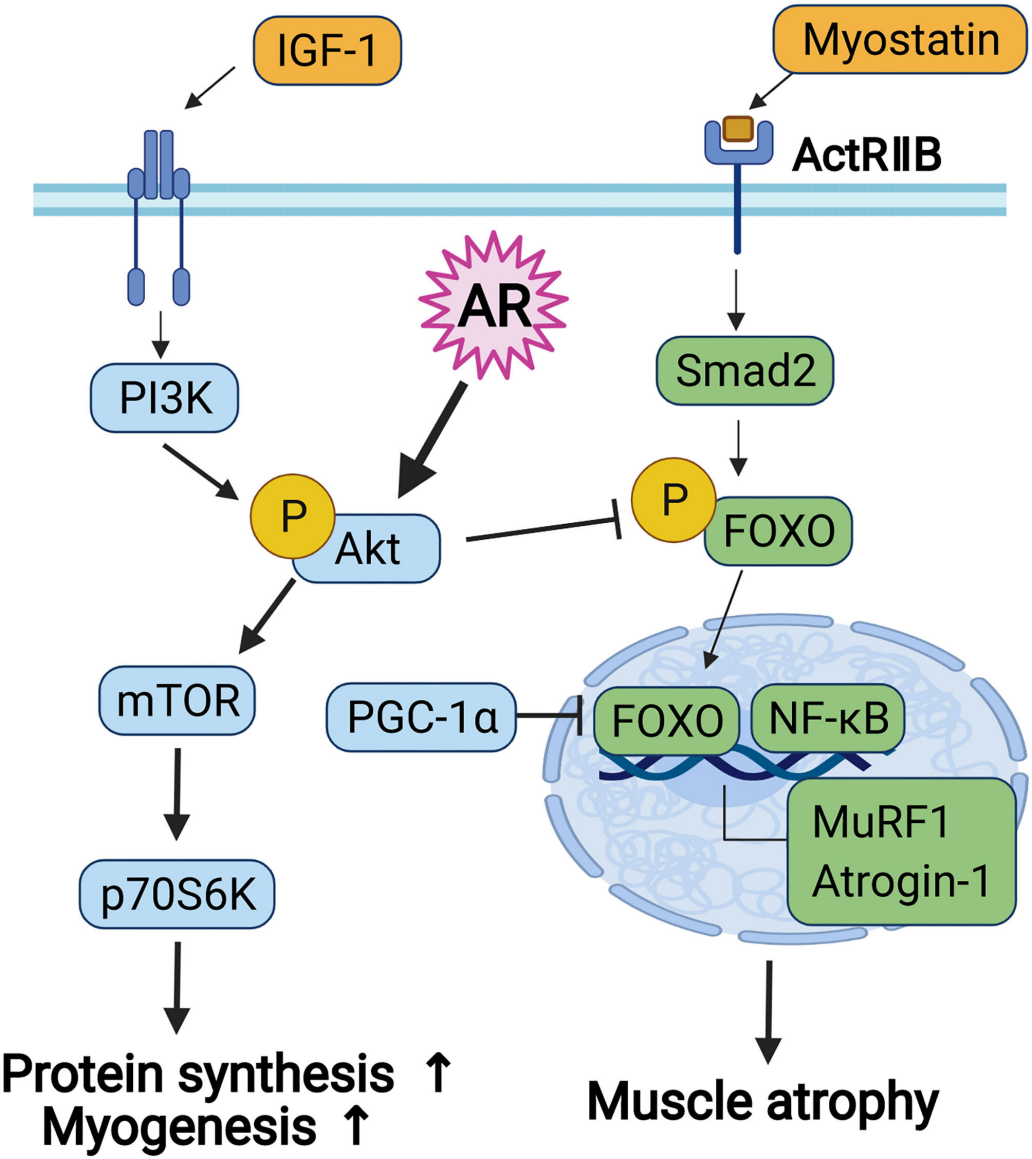
The diagram for the signaling pathway concerned to myoblast differentiation and protein metabolism.

Myoblast differentiation is initiated by the proliferation of myoblast and the subsequent cell cycle arrest, followed by a differentiation program, such as the expression of muscle-specific genes and the formation of multinucleated myofibers by myoblast fusion (43). Among diverse signaling pathways implicated in myoblast differentiation, Akt plays important roles in the induction of myoblast differentiation and muscle protein synthesis associated with myotube hypertrophy (24). Akt is a serine/threonine-protein kinase and is activated during myoblast differentiation. Overexpression of Akt promotes myogenic differentiation, whereas a dominant-negative form of Akt inhibits Akt activation and prevents myotube formation. In addition, ectopic expression of a constitutively active form of Akt can recover the inhibition of myoblast differentiation through inhibition of the PI3K (44). In addition, Akt plays a key role in PI3K/Akt/mTOR signaling and contributes to the regulation of energy metabolism and protein synthesis. The two major downstream target proteins of Akt/mTOR signaling regulating skeletal muscle hypertrophy are the p70S6K and the eIF4E-binding protein 1 (4EB-BP1), leading to upregulation of protein synthesis. The FoxO transcription factors regulate the activation of muscle-specific E3-ubiquitin ligases, MuRF1, and Atrogin-1 (22, 37). Especially, Akt/mTOR signaling enhances net protein accumulation by suppression of FoxO transcription factors and muscle-specific ubiquitin ligases implicated in protein catabolism (15, 16, 22). Akt induces the phosphorylation of FoxO transcription factors, resulting in inhibition of transcriptional functions of FoxOs through the exclusion of phosphorylated FoxO proteins from the nucleus. The Akt-mediated control of FoxO transcriptional functions is involved in the differentiation process of myoblasts (45). In addition to protein metabolic imbalance, the declined regenerative capacity of muscle stem cells is also tightly associated with the loss of muscle mass and function during aging or various muscle diseases (10). Our current results support that AR might promote the regenerative capacity of muscle stem cells and protein synthesis dependent on Akt, contributing to enhanced muscle mass and strength (Figure 6).

Peroxisome proliferator-activated receptor gamma coactivator-1 alpha plays a central role in a regulatory network governing the transcriptional control of mitochondrial biogenesis. PGC-1α evokes the expression of genes associated with exercise, such as genes involved in mitochondrial biogenesis, stimulation of fatty acid oxidation, angiogenesis, and resistance to muscle atrophy (46). The NADH and SDH participate in citric acid cycle of mitochondria. NADH-TR reaction is a histochemical reaction used for evaluation of the space between the myofibrils referred to as the inter-myofibrillary matrix. This reaction is catalyzed by enzymes in the mitochondria (NADH-dehydrogenase) or in the endoplasmic reticulum (NADH cytochrome b5 reductase). SDH is a reaction specific for mitochondria that are typically used to screen for mitochondrial myopathies. While NADH-TR reactivity implies activation of enzymes in the mitochondria or endoplasmic reticulum, SDH reactivity implies activation of enzymes specific to the mitochondria. Glucose 6-phosphate dehydrogenase (GPDH) is a marker for reductive biosynthesis; Mitochondrial GPDH is an integral component of the mitochondrial respiratory chain and functions as the rate-limiting step in the glycerophosphate (GP) shuttle. Mitochondrial GPDH expression was significantly increased over the course of C2C12 myoblast differentiation, with an expression profile similar to that of Myogenin and MHC (47–49). Specifically, it regulates myogenic markers and myoblast differentiation by controlling mitochondrial biogenesis via CaMKKβ/AMPK. Mitochondrial GPDH^−/−^ attenuates skeletal muscle regeneration *in vitro* and *in vivo*, while overexpression of mitochondrial GPDH ameliorates dystrophic pathology in mdx mice. Mitochondrial biogenesis in regulating myogenic differentiation pays attention as a potential therapeutic target for ameliorating muscle regeneration impairment and muscle pathology. In our results, AR induces the increase of both oxidative (MyhIIa) and glycolytic (MyhIIb) myofibers and enhances the expression of muscle metabolic genes, including mitochondrial components.

The fruit of aronia consists of a variety of ingredients, such as anthocyanin, flavonoids, ursolic acid, acetylursolic acid, and oleanolic acid (28, 50, 51). These components have been implicated in the increase of muscle mass, fast, and slow muscle fiber size and muscle strength (52–54). The treatment with ursolic acid, one of the main components of aronia, increases muscle mass in mice through enhancing muscle insulin/IGF-I signaling and inhibiting atrophy-associated muscle mRNA expression (52). Since Akt is the major downstream signaling component of IGF-1, this is in consistent with our current data. Although the functional aspect is not addressed, another study has proposed that the effect of aronia might be exerted through mTORC1 activation in response to resistance exercise without increasing muscle protein synthesis (50). Further studies are required to define the regulatory mechanisms by which aronia exerts the beneficial effect on muscle mass and strength. Our current study also demonstrates that aronia treatment upregulates the activities of both oxidative and glycolytic metabolic enzymes with increased expression of mitochondrial genes and glucose metabolism-related genes (Figure 5).

## Conclusions

Our study demonstrates that AR promotes myogenic differentiation through Akt activation and protects the DEX-induced myotube atrophy through Akt activation that in turn represses the expression of muscle-specific ubiquitin ligases. In addition, AR treatment improves muscle mass and strength with increased expression of both glycolytic and oxidative myofibers with upregulated muscle-specific genes and muscle metabolic genes. Thus, given the current lack of therapies for skeletal muscle atrophy, AR might be a promising potential as a nutraceutical remedy to intervene in muscle weakness and atrophy.

## Data Availability Statement

The original contributions presented in the study are included in the article/supplementary material, further inquiries can be directed to the corresponding author/s.

## Ethics Statement

All animal experiments were approved by the Institutional Animal Care and Research Advisory Committee at Sungkyunkwan University School of Medicine (SUSM) and complied with the regulations of the Institutional Ethics Committee.

## Author Contributions

S-JL and G-UB conceptualized the project, designed the experiments, wrote the manuscript, and supervised the project. C-EY, H-KS, TAV, MWN, and SA performed the experiments. H-KL, KHK, and J-SK analyzed the results and performed the statistical analysis. All authors contributed to the article and approved the submitted version.

## Funding

This research was supported by the National Research Foundation Grant funded by the Korean Government (MSIP) (NRF-2019R1A2C2006233).

## Conflict of Interest

H-KS, TAV, and S-JL are employed by AniMusCure INC. The remaining authors declare that the research was conducted in the absence of any commercial or financial relationships that could be construed as a potential conflict of interest.

## Publisher's Note

All claims expressed in this article are solely those of the authors and do not necessarily represent those of their affiliated organizations, or those of the publisher, the editors and the reviewers. Any product that may be evaluated in this article, or claim that may be made by its manufacturer, is not guaranteed or endorsed by the publisher.
